# Supplementary material for: The Homoleptic Curcumin–Copper Single Crystal (ML2): A Long Awaited Breakthrough in the Field of Curcumin Metal Complexes
Source: Molecules. 2023 Aug 12;28(16):6033. doi: 10.3390/molecules28166033 (PMC10458717; doi:10.3390/molecules28166033)
Supplement: Supplementary file 1 [file molecules-28-06033-s001.zip › molecules-2501721-supplementary.pdf]

# *Supplementary Materials*

# The Homoleptic Curcumin–Copper Single Crystal (ML$_2$): A Long Awaited Breakthrough in the Field of Curcumin Metal Complexes

**Antonino Arenaza-Corona [1,†], Marco A. Obregón-Mendoza [1,†], William Meza-Morales [2], María Teresa Ramírez-Apan [1], Antonio Nieto-Camacho [1], Rubén A. Toscano [1], Leidys L. Pérez-González [1], Rubén Sánchez-Obregón [1] and Raúl G. Enríquez [1,*]**

[1] Instituto de Química, Universidad Nacional Autónoma de México, Ciudad de México 04510, Mexico; antoninoarenaza03@gmail.com (A.A.-C.); obregonmendoza@yahoo.com.mx (M.A.O.-M.); mtrapan@yahoo.com.mx (M.T.R.-A.); camanico2015@yahoo.com (A.N.-C.); toscano@unam.mx (R.A.T.); leidyslaura92@gmail.com (L.L.P.-G.); rubens@unam.mx (R.S.-O.)

[2] Department of Chemical Engineering, University of Puerto Rico-Mayaguez, Mayagüez, PR 00680, USA; willy_meza_morales@hotmail.com

* Correspondence: enriquezhabib@gmail.com; Tel.: +52-55562-24404

† These authors contributed equally to this work.

TABLE OF CONTENTS


1. **Single crystal X-ray diffraction** .................... 3
2. **Electrospray ionization** .................... 5
3. **EPR and CP/MAS** .................... 8
4. **IR (ATR)** .................... 9
5. **UV-Vis** .................... 11
6. **PXRD** .................... 12
7. **Fingerprints plots** .................... 13
8. **Cytotoxicity** .................... 14
9. **Antioxidant activity** .................... 14

## 1. Single crystal X-ray diffraction

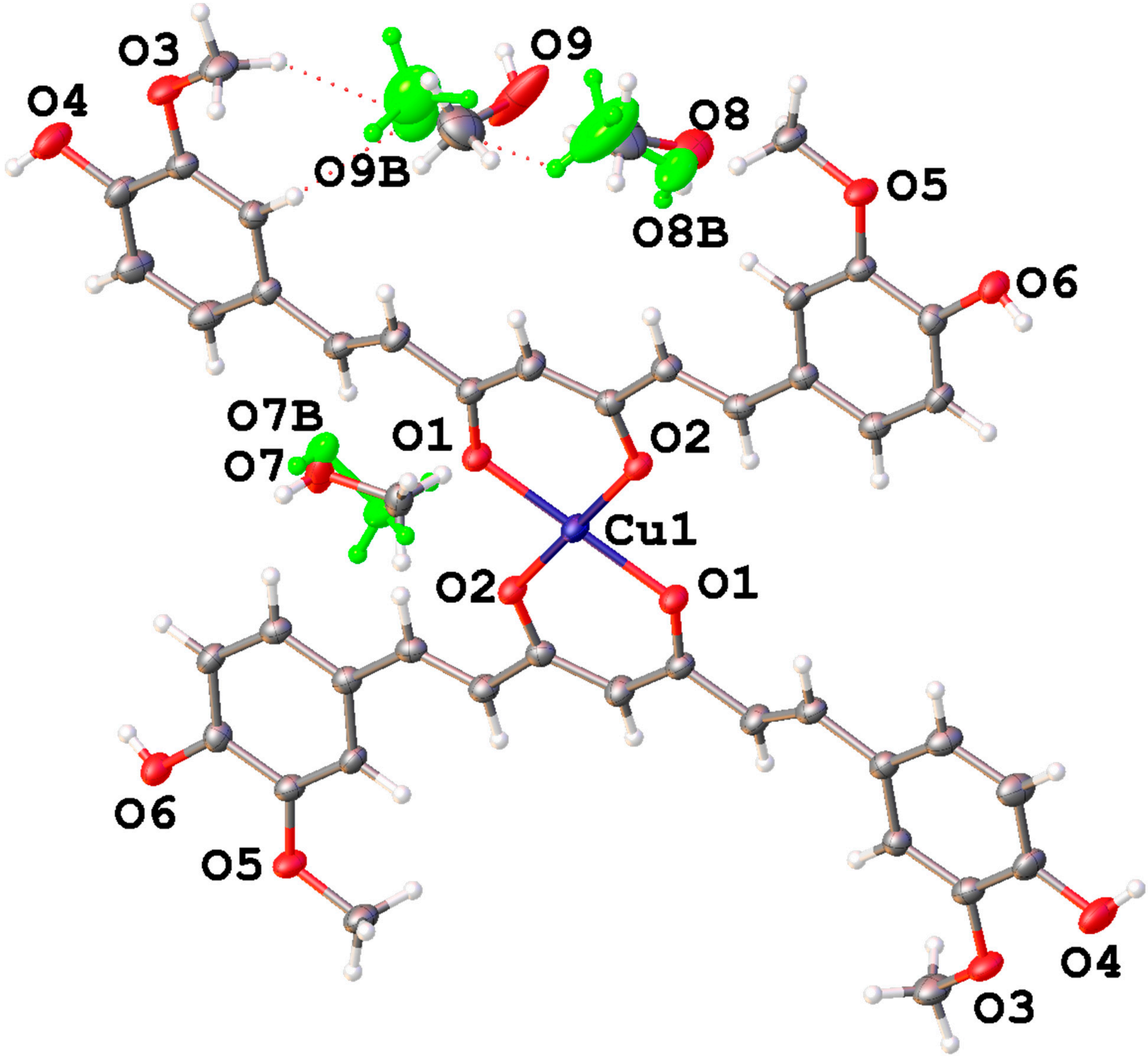


**Figure S1.** The molecular structure of complex **1** including molecules of solvent (methanol) and the disordered position of methanol (green ellipsoids).

**Table S1.** Crystallographic data of complex **1**.

| Empirical Formula | $C_{42}H_{38}O_{12}Cu \cdot 5MeOH$ |
|---|---|
| Formula weight | 958.47 |
| Temperature/K | 130(2) |
| Crystal system | *triclinic* |
| Space group | P-1 |
| a/Å | 8.3372(7) |
| b/Å | 8.7677(4) |
| c/Å | 16.3052(12) |
| α/° | 75.664(5) |
| β/° | 84.240(6) |
| γ/° | 84.636(5) |
| Volume/Å$^3$ | 1146.02(14) |
| Z | 1 |
| $_{Scalc}$ g/cm$^3$ | 1.389 |
| μ/mm$^{-1}$ | 0.551 |
| F(000) | 505.0 |
| Crystal size/mm$^3$ | 0.33 × 0.13 × 0.06 |
| Radiation | MoK$\alpha$ ($\lambda$ = 0.71073) |
| 2Θ range for data collection/° | 6.852 to 59.284 |
| Index ranges | $-11 \leq h \leq 11$, $-12 \leq k \leq 11$, $-22 \leq l \leq 22$ |
| Reflections collected | 25565 |
| Independent reflections | 5848 [$R_{int}$ = 0.0466, $R_{sigma}$ = 0.0476] |
| Data/restraints/parameters | 5848/120/377 |
| Goodness-of-fit on F$^2$ | 1.047 |
| Final R indexes [I>=2σ (I)] | $R_1$ = 0.0498, $wR_2$ = 0.1160 |
| Final R indexes [all data] | $R_1$ = 0.0706, $wR_2$ = 0.1293 |

## 2. Electrospray ionization

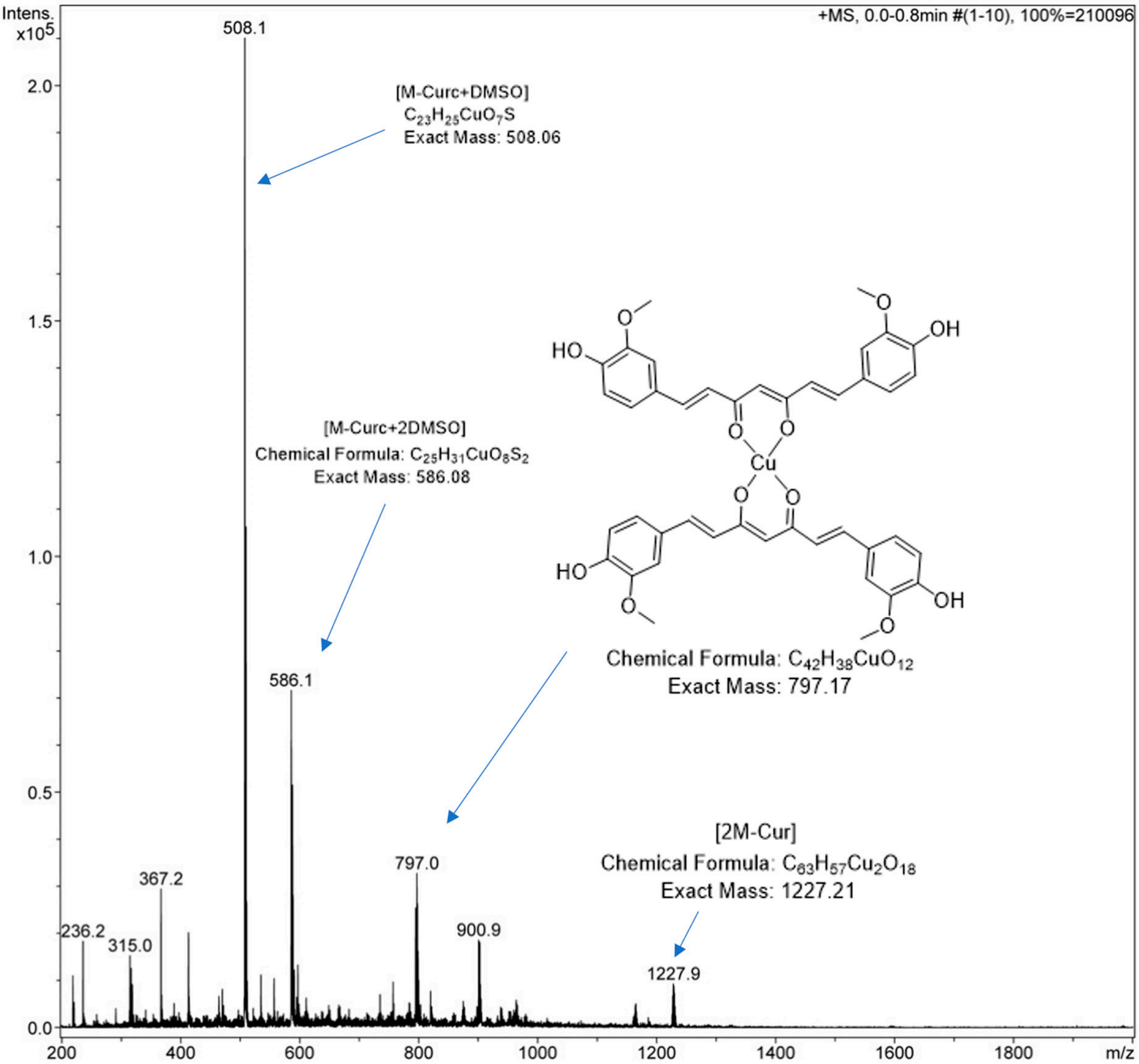


**Figure S2.** Mass spectrum of complex **1** (dark brown solid) by ESI$^+$ previously dissolved in DMSO.

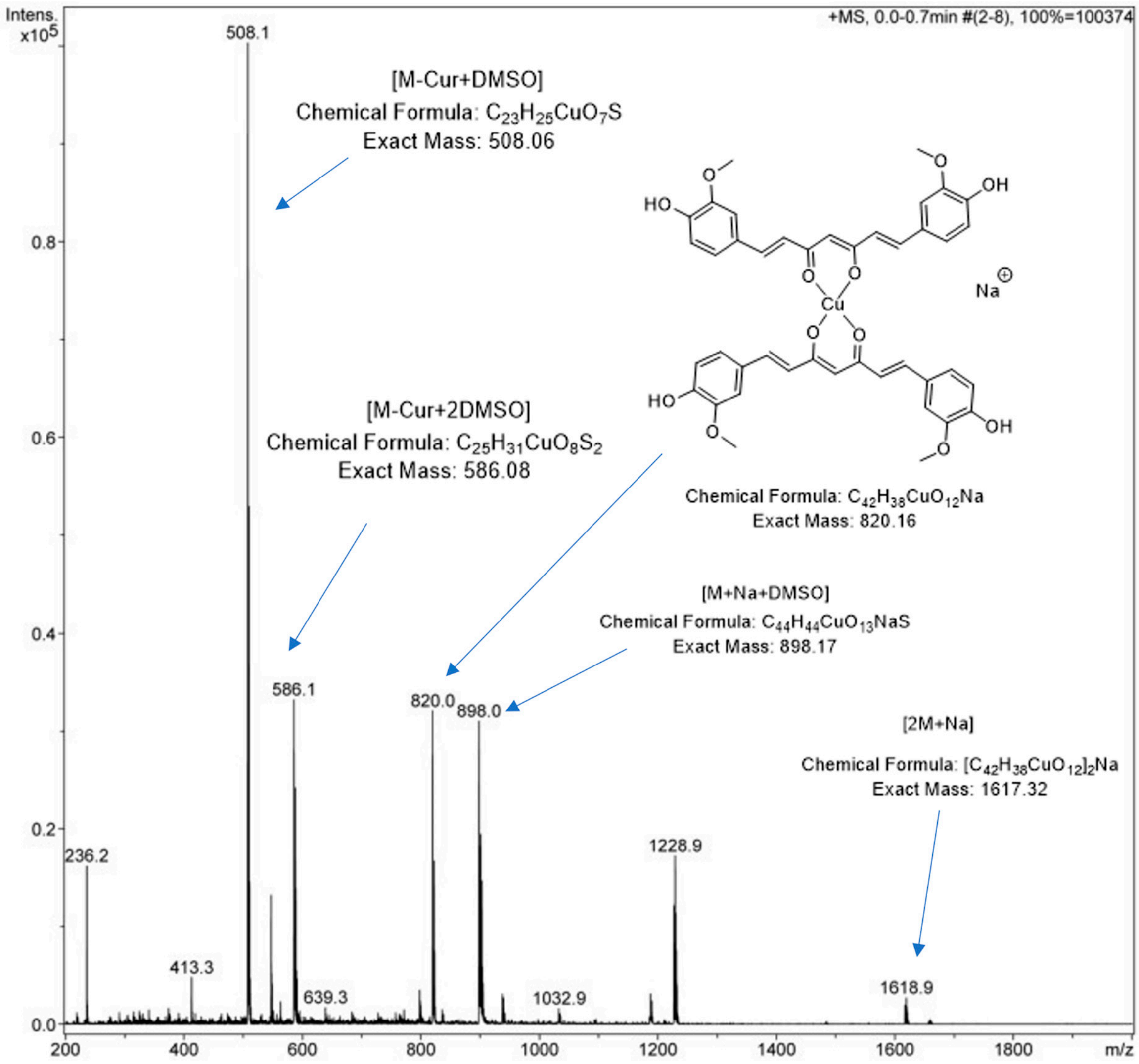


**Figure S3.** Mass spectrum of complex **1** (brilliant brown solid) by ESI+ previously dissolved in DMSO.

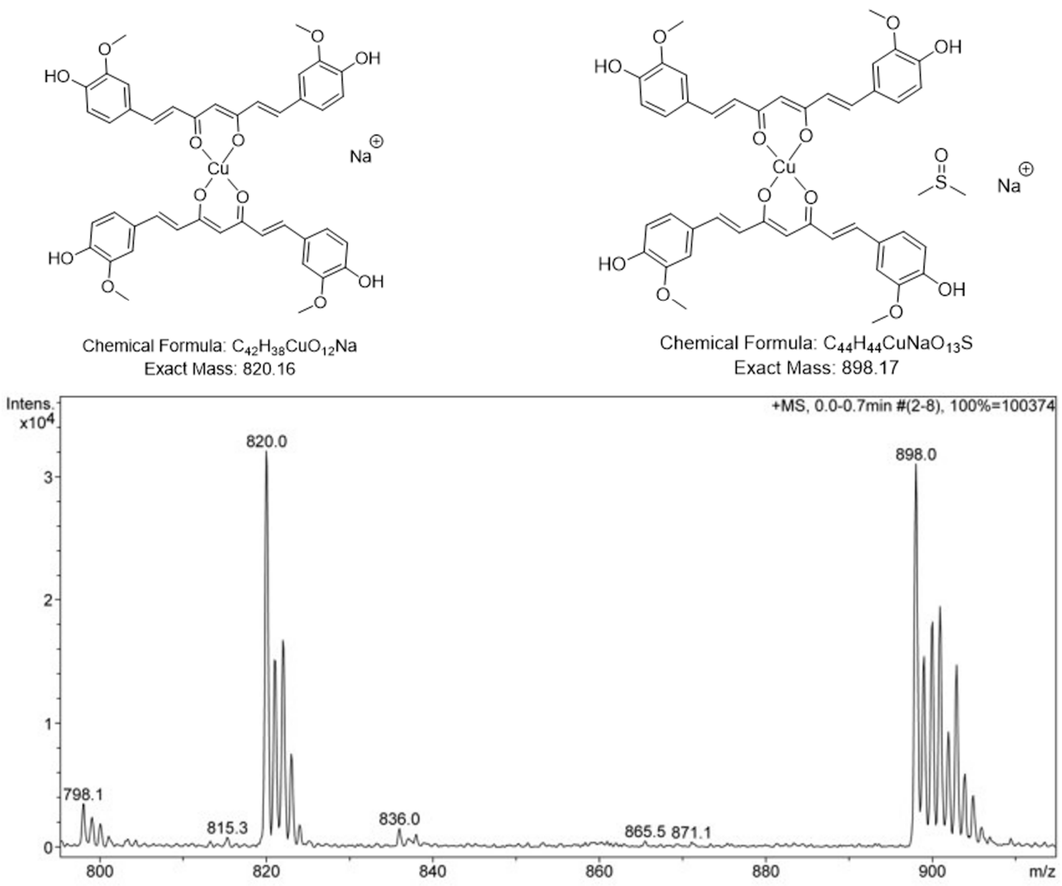


**Figure S4.** Amplification of Mass spectrum of complex **1** (brilliant brown solid) by ESI$^+$ previously dissolved in DMSO.

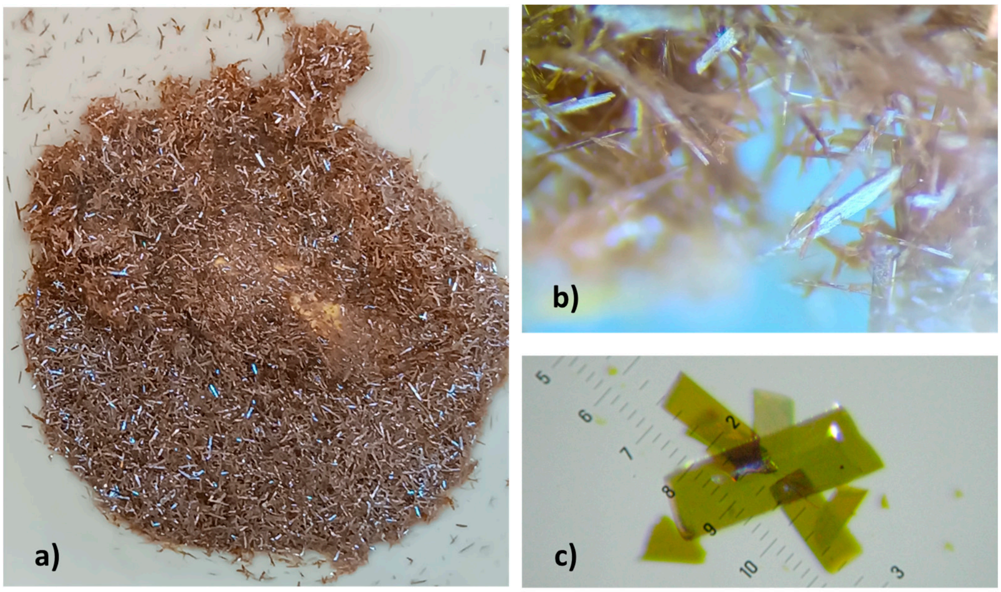


**Figure S5**. Grow process of a monocrystal of complex **1**: (**a**) Recrystallization of the filtrate and filtration in a Hirsch funnel, (**b**) amplified view of crystals, and (**c**) Monocrystals obtained after slow evaporation in methanol.

## 3. EPR and CP/MAS

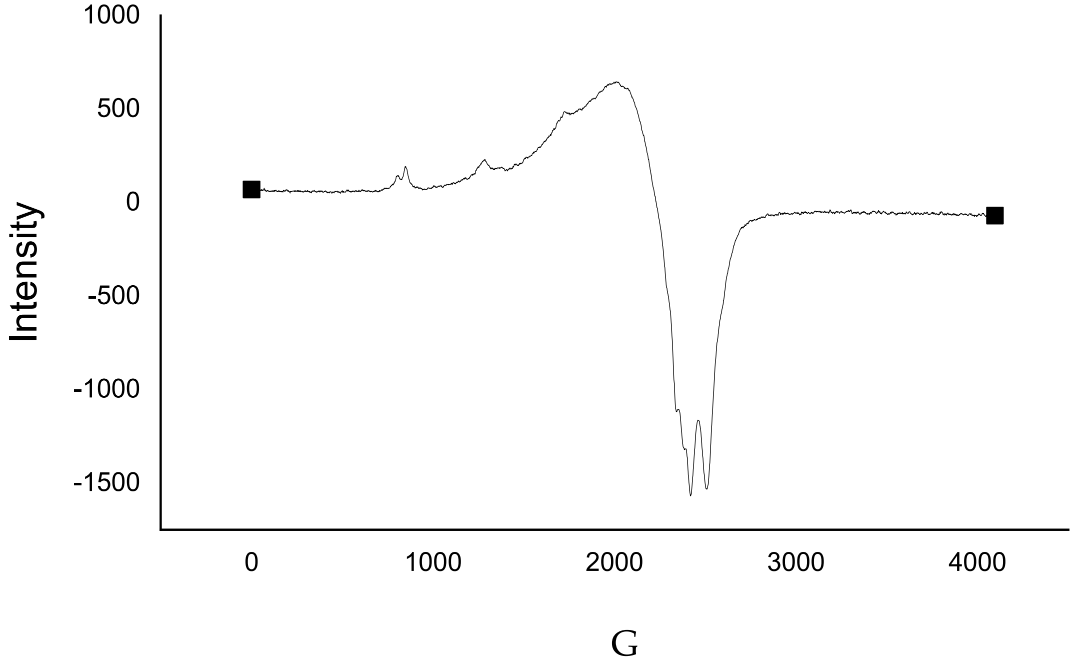


**Figure S6.** Solid state Electron Paramagnetic Resonance of amorphous material (dark brown solid).

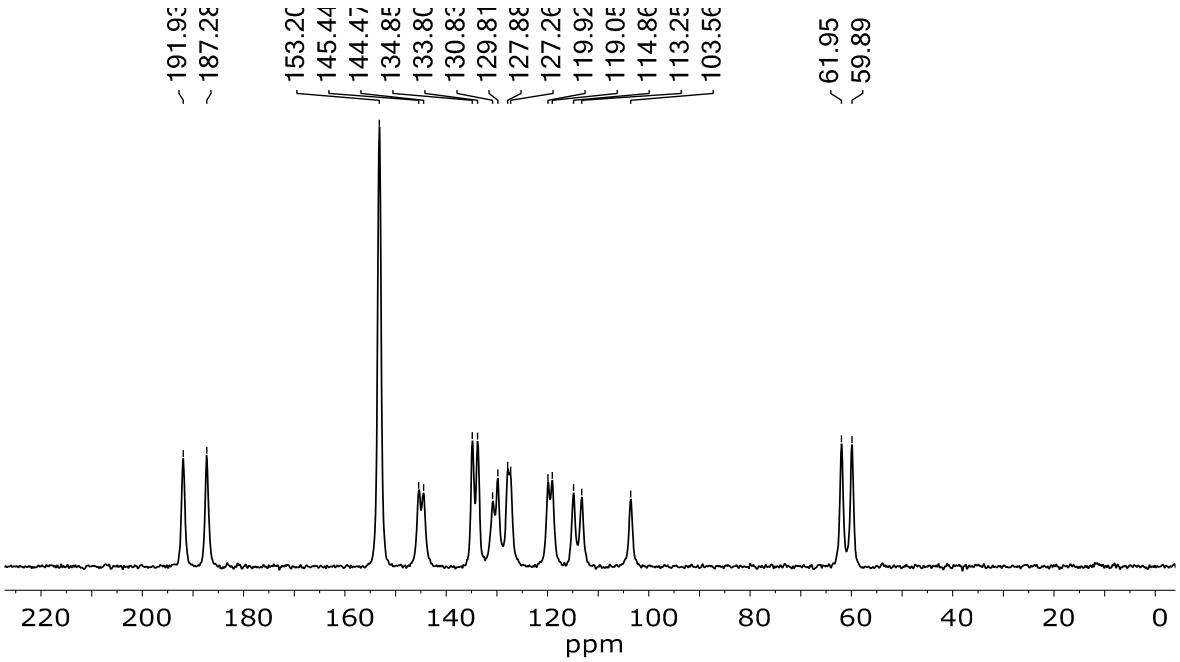


**Figure S7.** $^{13}C$ CP/MAS spectrum of curcumin.

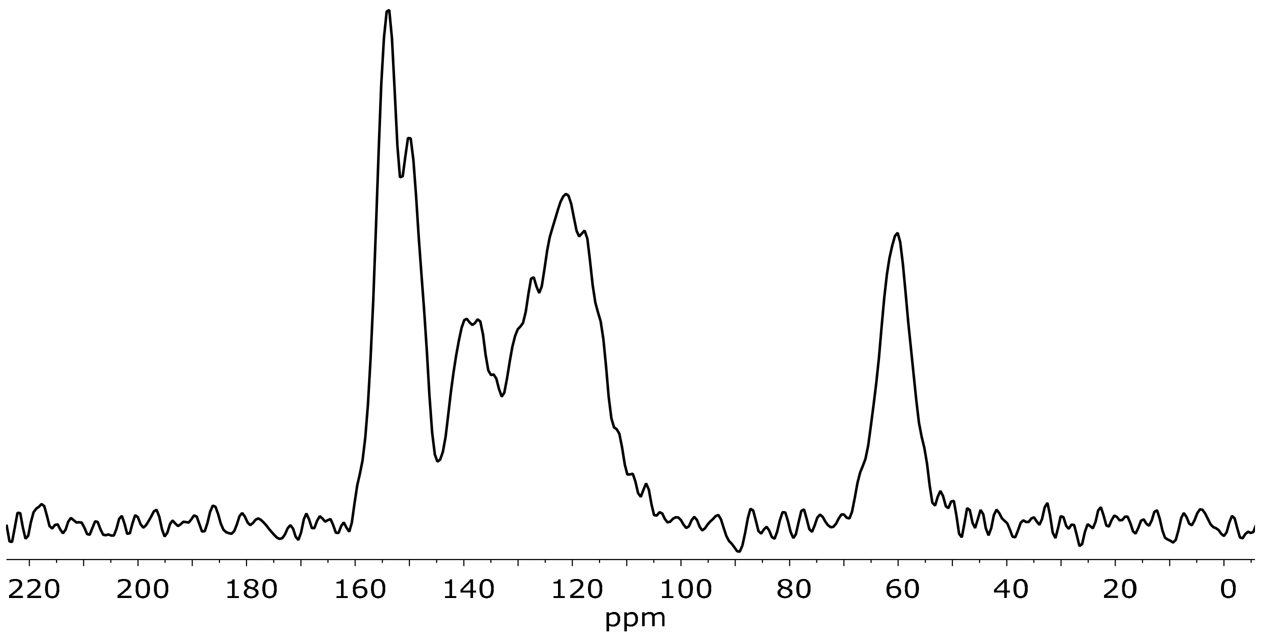


**Figure S8.** $^{13}C$ CP/MASS spectrum of complex (**1**).

## 4. IR (ATR)

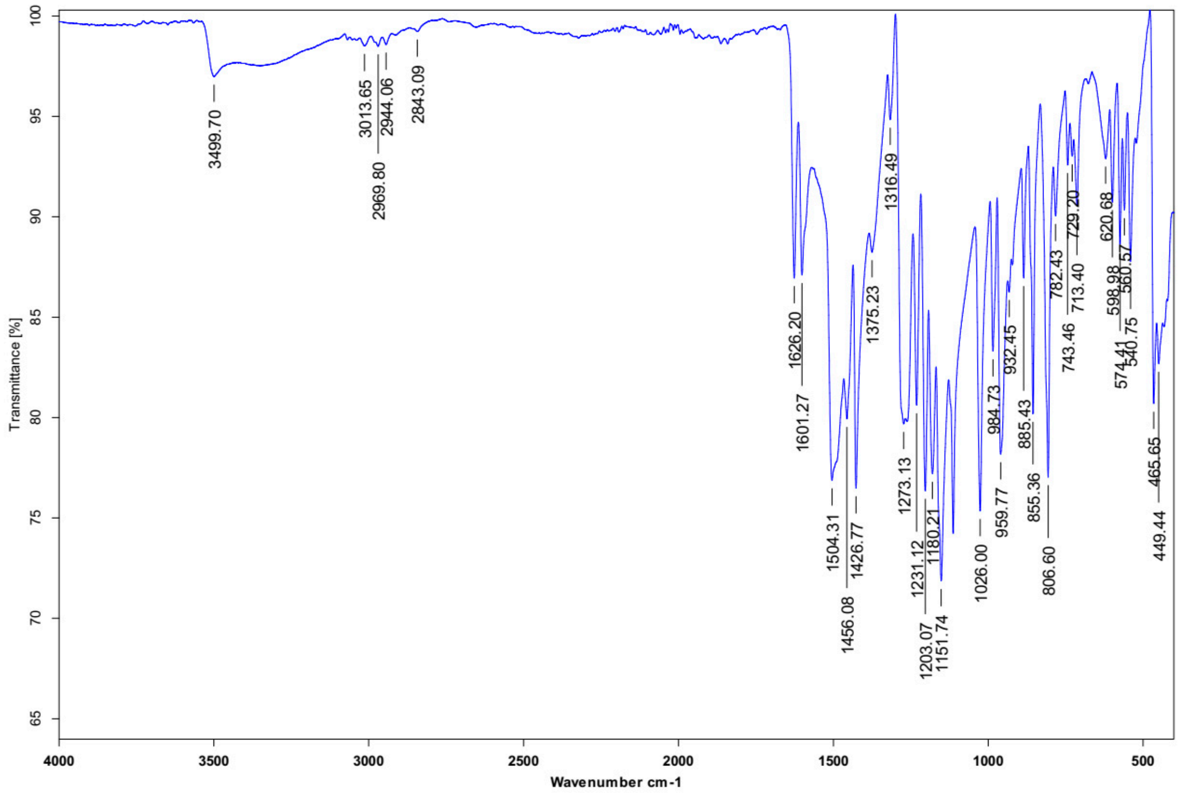


**Figure S9.** IR-ATR spectrum of free curcumin.

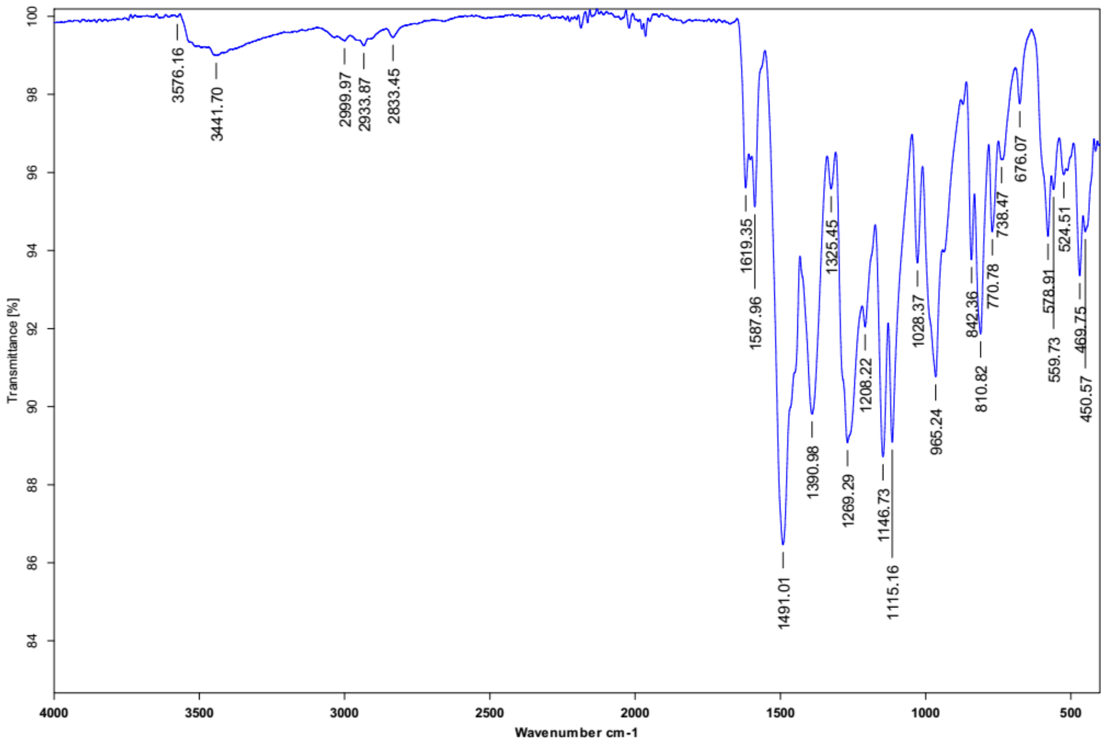


**Figure S10.** IR-ATR spectrum of complex (**1**) dark brown solid.

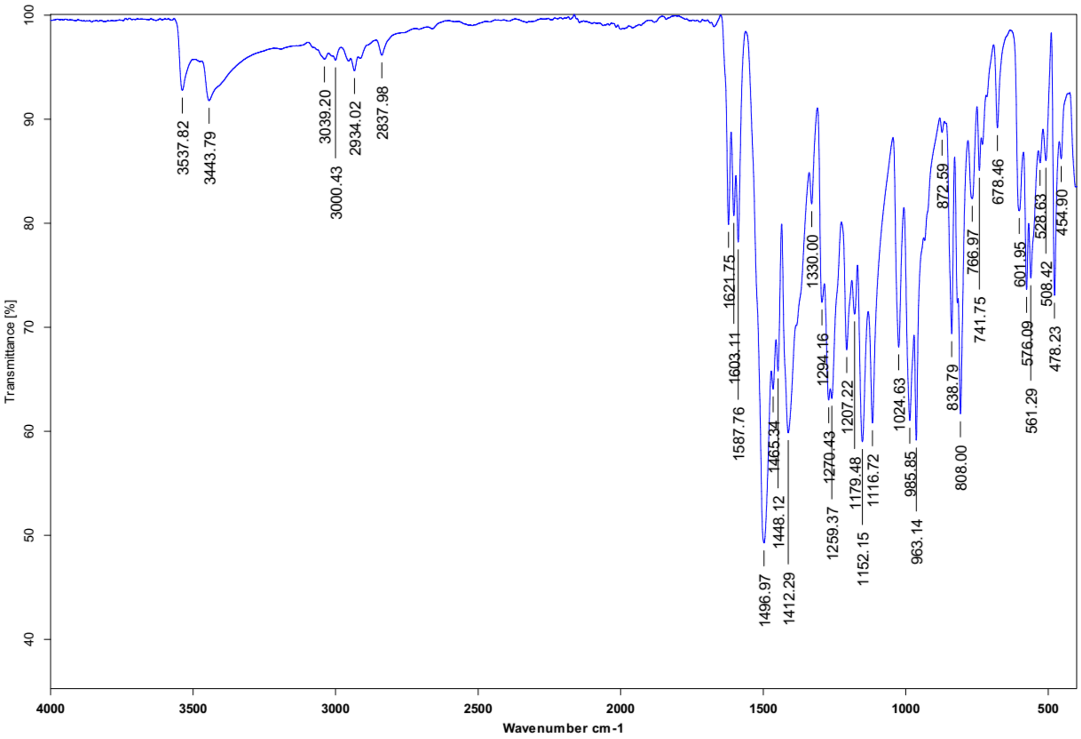


**Figure S11.** IR-ATR spectrum of complex (**1**) brilliant brown crystalline solid.

## 5. UV-Vis

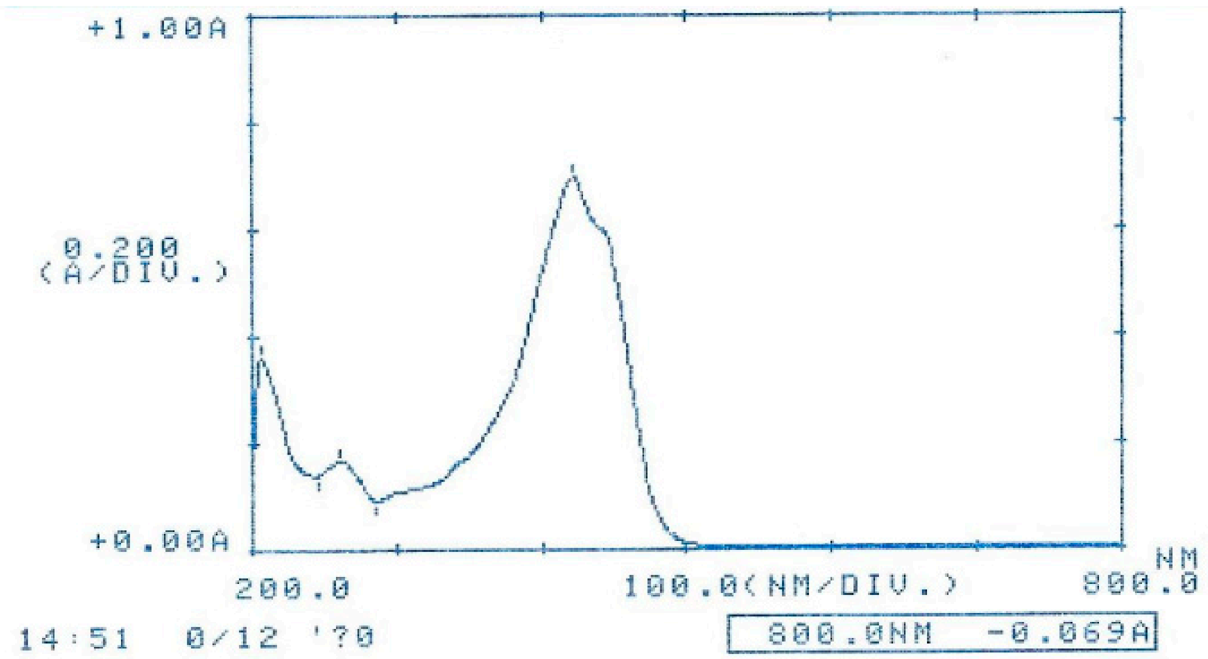


**Figure S12.** UV-Vis spectrum of complex (**1**) dark brown solid (REF: Methanol, Max= 422 nm).

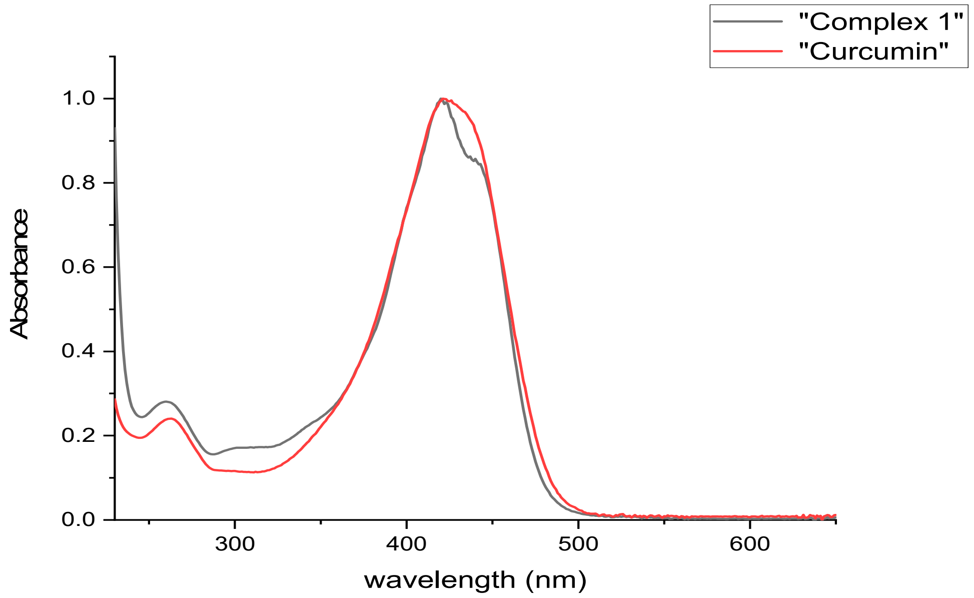


**Figure S13.** UV-Vis spectra of complex (**1**) brilliant brown crystalline solid (REF: Methanol, Max= 421 nm) and free curcumin (REF: Methanol, Max= 420 nm).

## 6. PXRD

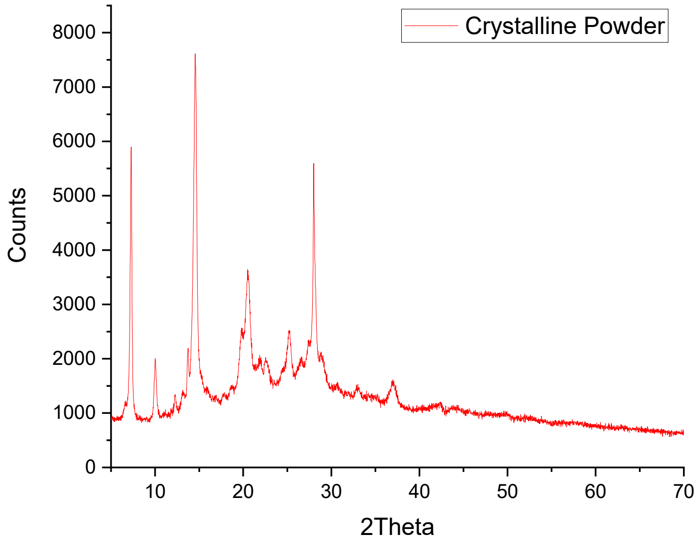


**Figure S14.** Powder XRD pattern of complex **1** (bright brown material).

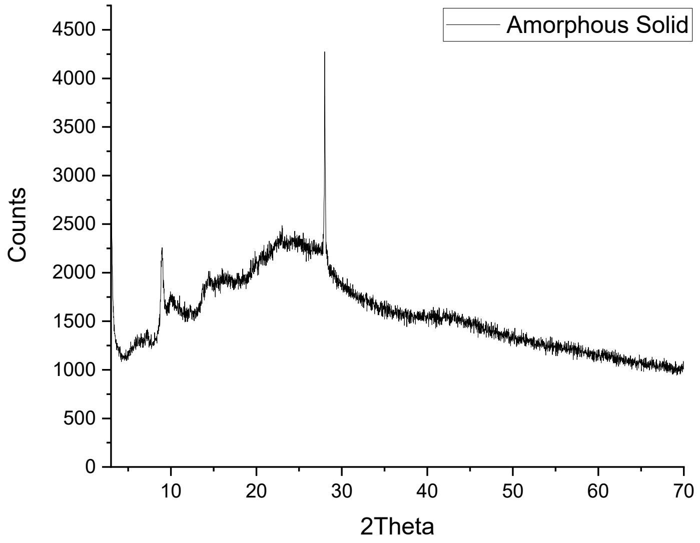


**Figure S15.** Powder XRD pattern of complex **1** (amorphous solid).

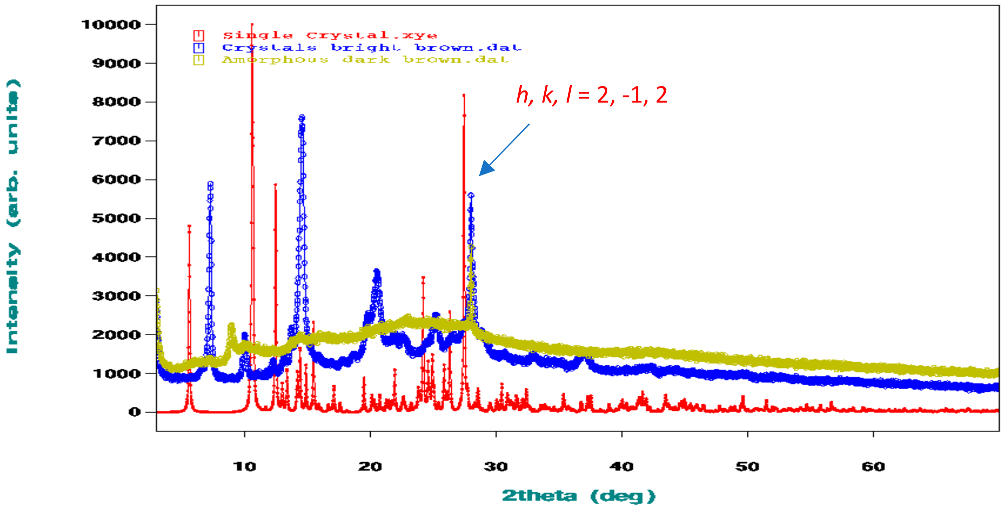


**Figure S16.** Comparison of PXRD patterns of complex **1** (red: simulated, 130K); blue: crystalline material; gold: amorphous material).

## 7. Fingerprints plots

| Contact | Fingerprint | Contact | Fingerprint |
|---|---|---|---|
| O···H/H···O 24.5% | 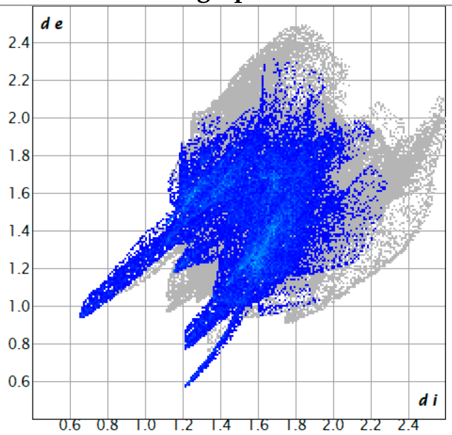 | C···H/H···C 17.8 % | 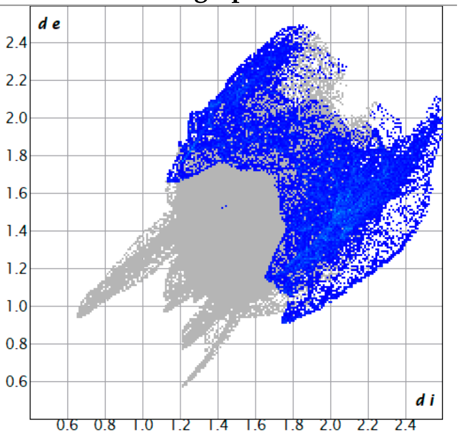 |
| H···H 44.2 % | 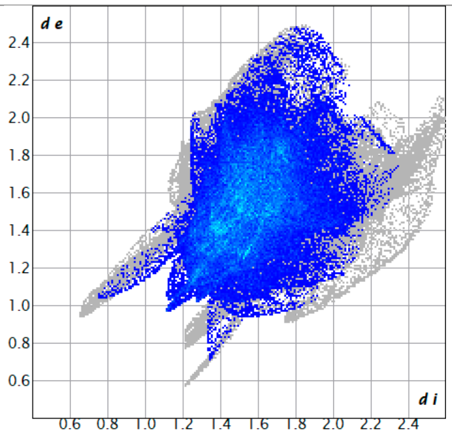 | C···C 7.4 % | 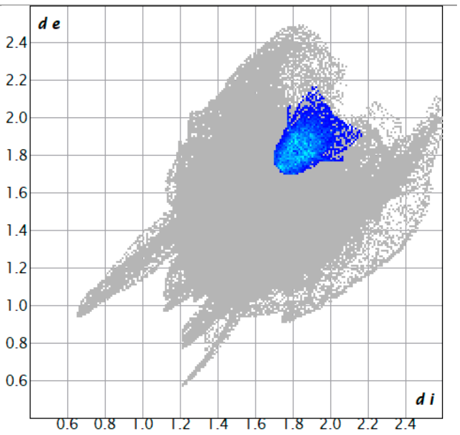 |

## 8. Cytotoxicity

**Table S2.** Growth inhibition percentages of cancerous and noncancerous (COS-7) cell lines by compounds (9.9 μM, 48 h).

| | Grow inhibition (%) by cell line | | | | | | |
|---|---|---|---|---|---|---|---|
| **Sample** | **U251** | **PC-3** | **K562** | **HCT-15** | **MCF-7** | **SKLU-1** | **COS 7** |
| **Curcumin** | NC | 22.7 | 20.81 | 10.5 | 1.30 | 5.8 | 5.05 |
| **Complex 1** | 35.43 | 29.3 | 62.87 | 18.8 | 0.55 | 26.1 | 20.35 |

Inhibition percentages of complex **1** relative to curcumin. Growth inhibition percentages of cancerous and noncancerous (COS-7) cell lines by compounds. U251 (human glioblastoma), PC-3 (human prostatic adenocarcinoma), K562 (human chronic myelogenous leukaemia), HCT-15 (human colorectal adenocarcinoma), MCF-7 (human mammary adenocarcinoma), cell lines were supplied by the National Cancer Institute (USA); and SKLU-1 (human lung adenocarcinoma) and COS-7 cell line (monkey African green kidney) were donated by the Cancer Institute of Mexico. *The results are the average of three runs.

## 9. Antioxidant activity

**Table S3.** Inhibition of Lipid Peroxidation on Rat Brain.

| **Sample** | **Concentration (mM)** | **TBARS (nmol/mg prot.)** | **Inhibition (%)** | **$IC_{50}$ (mM)** |
|---|---|---|---|---|
| **Complex 1** | Basal | 0.319±0.051 | | |
| | Control | 9.038±0.512 | | |
| | 0.18 | 7.759±0.548 | 14.27±2.02 | 1.24±0.08 |
| | 0.32 | 7.242±0.492* | 19.98±1.14* | |
| | 0.55 | 6.230±0.488** | 31.24±1.80** | |
| | 0.99 | 4.987±0.315** | 44.81±1.88** | |
| | 1.76 | 3.239±0.342** | 63.61±5.30** | |
| | 3.12 | 1.766±0.525** | 79.69±6.78** | |
| | 5.55 | 0.622±0.053** | 93.02±0.91** | |

| | | | | |
|---|---|---|---|---|
| | 9.87 | 0.353±0.072** | 96.16±0.58** | |
| **Curcumin** | Basal | 0.319±0.051 | | |
| | Control | 9.038±0.512 | | |
| | 0.18 | 8.345±0.412 | 7.58±1.12 | 3.03±0.15 |
| | 0.32 | 8.136±0.403 | 9.88±1.97 | |
| | 0.56 | 7.542±0.462 | 16.6±0.39 | |
| | 1 | 6.932±0.418** | 23.32±1.34** | |
| | 1.78 | 6.100±0.322** | 32.48±0.39** | |
| | 3.16 | 4.267±0.093** | 52.46±3.01** | |
| | 5.62 | 2.289±0.343** | 74.11±5.04** | |
| | 10 | 0.881±0.286** | 89.84±3.81** | |
| **BHT** | Basal | 0.268±0.053 | | |
| **(n=3)** | Control | 7.384±0.630 | | |
| | 0.56 | 6.098±0.353 | 16.64±2.86 | 1.22±0.44 |
| | 0.75 | 5.559±0.294* | 23.92±2.69* | |
| | 1 | 4.457±0.283** | 37.14±7.44** | |
| | 1.33 | 3.228±0.572** | 53.59±8.93** | |
| | 1.78 | 1.315±0.489** | 81.59±6.89** | |
| | 2.37 | 0.487±0.075** | 93.16±1.16** | |

Homogenised in: PBS; Vehicle: DMSO; Experiment: Curve; Peroxidation: induced with $FeSO_4$ 10 mM, Incubation time: 1 h; EDTA: 2 mM. The values represent the average of three independent experiments ± standard error of the media $(\bar{x} \pm ES)$. Data were subjected to an analysis of variance (ANOVA) followed by a Dunnett pass to isolate groups with significant differences. The values of $p \leq 0.05$ (*) and $p \leq 0.01$ (**) were considered as significant differences with respect to control.

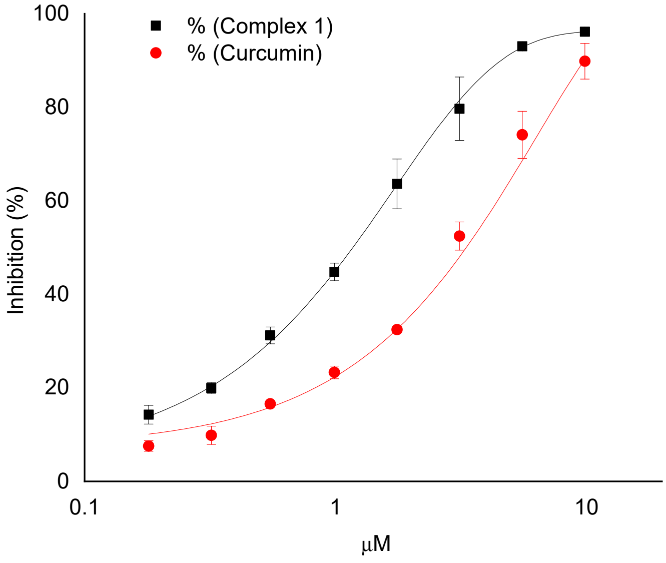


**Figure S17.** Inhibition curve of Lipid Peroxidation on Rat Brain.

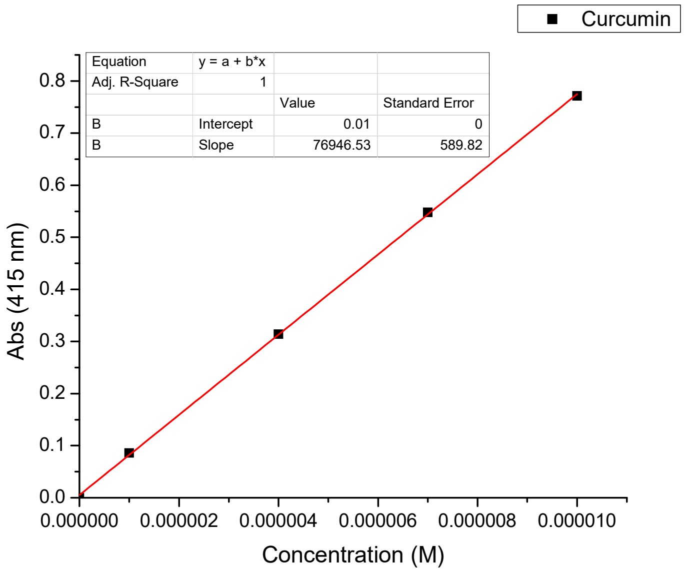


**Figure S18.** Adj. R-Square of Curcumin in 1-Octanol.

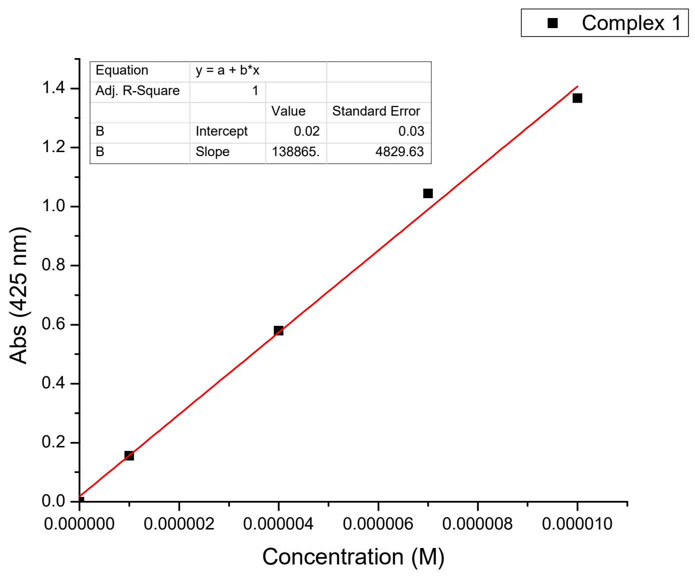


**Figure S19.** Adj. R-Square of **Complex 1** in 1-Octanol.
